# Practical Experience in Typhoid Fever as It Occurs in Alabama

**Published:** 1888-07

**Authors:** Frank Prince

**Affiliations:** Bessemer, Ala., Ex-Vice-President and Senior Counselor of the Medical Association of the State of Alabama


### PRACTICAL EXPERIENCE IN TYPHOID FEVER AS IT OCCURS IN ALABAMA.*

---

BY FRANK PRINCE, M. D., BESSEMER, ALA.,

Ex-Vice-President and Senior Counselor of the Medical Association of the State of Alabama.

---

An honest opinion is the outgrowth of facts and knowledge, either obtained from a foreign source or home surroundings, and is entitled to investigation, no matter how erroneous it may seem to others. Man is a peculiar animal, has individually his peculiar way of seeing, hearing, thinking, and on this his acting more or less depends; and while we differ in taste, in morals and appreciation, why may we not differ intellectually without being compromised in the eyes of another?

When Harvey advanced his theory of the circulation of the blood, he was called a fool; and in the days of Aristotle astronomers held the position that all the planets, sun not excepted,

*Partially read before and reprinted from the Transactions of the Medical Association of the State of Alabama.

revolved around the earth, and the sun, in its biblical sense, was created alone to give light by day and the moon by night. The main thought of this theory was impressed upon Copernicus, that of revolution, and he advanced the theory that rules at the present day—that the sun was fixed, and the earth and all the other planets revolved around it, and for this Martin Luther said of him, "the old fool wants to turn the world upside down; the Bible tells us Joshua commanded the sun to stand still and it obeyed." One generation evolves facts or points for the development of facts for the generation that is to follow.

My subject carries me back over a journey of thirty-seven years, and if on it I have gathered any flowers by the wayside, you are welcome to them to-day.

Typhoid fever is a disease peculiar to no clime, section or locality, being found upon the highest hills and lowest vales where a human being can dwell. In the mountains it is often seen as a visitor, and as often in the swamps, where the ague loves to revel. In the newest countries, as well as in the oldest—in the forest covered as well as in the land divested of timber. It was once thought that it had respect for the oldest and youngest person and never attacked them; but there are years and peculiar localities where these seem to be its choicest victims, as was the case with us in 1883. But between the ages of ten and thirty years more cases occur than at any other period of life. It does not always show itself under the same guise. It may come on suddenly, and then the danger is less, or slowly and insidiously, and then the form of fever is more violent. And under three slightly differing forms have I seen it, at the same time, in the same section of country, but on different subjects.

As a matter of convenience to myself, I have divided it into three grades as follows:

Typhoid Mitior;

Constipated Typhoid Fever; and

Typhoid Gravior.

The symptoms that manifest themselves in typhoid mitior are headache—sometimes bleeding at the nose with flushed face—a sense of weariness; bowels sore and tender upon pressure, swollen and relaxed; skin tight over the abdomen; palms of the hands dry and rough; pulse with a cramped beat, 100 to 120 per minute; temperature 100° to 102°; listless, somewhat indifferent to what is passing around him, but will answer correctly when spoken to; will put out his tongue when told to do so, but often has to be told "that will do," or to take it back; tongue sometimes tremulous, dry-pointed with red edges, with light coat to the front, dark-brown centre, getting darker towards the fauces. And then, at other times, it fills the mouth with tooth-marks upon it; slightly dry, hacking cough from beginning of attack; eruptions at or near the umbilicus. These symptoms often continue until the 27th day, when the fever disappears.

But in a mountainous region of country, in addition, there are symptoms of intermittent type that might mislead, but in every case the true typhoid symptoms are never wanting, and as the case progresses, these override all others, and the intermittents are all lost or absorbed in the more violent form of fever.

All cases of typhoid fever have not the same peculiar symptoms, but there is considerable diversity of symptoms, more or less so, as there are differences in constitutions, atmospheric influences and locality. In constipated typhoid fever, most commonly the premonitory symptoms are protracted for some days. The patient feels unwell, with lassitude, yawning, loss of appetite; lies upon his back, knees drawn up. The pulse is hard, corded, ranging from 110 to 130; ringing in the ears, dark spots floating before the eyes. The tongue is sometimes large, full and pale, with tooth-marks upon it, then again, brown and dry, red, sleek and glazed. Teeth covered with sordes, urine scanty, bowels constipated, stupor, distended abdomen, with tympanitis, and occasional hemorrhage from the nose. Temperature from 100° to 104°. No intermission of fever, but from 2 p. m. to 10 a. m. fever lower. Patient complains of no pain, and when asked how he feels, generally answers, better. Headache is a common symptom, and long-continued headache denotes severe inflammation of the glands of Peyer, and should warn us of the greater danger of the near approach of hemorrhage.

Typhoid gravior is when all the symptoms are greatly aggravated. Great soreness and tenderness of the bowels and thin, dark-colored discharges often involuntary action of the bowels; dry, hacking cough, great dullness of expression, stupor, no disposition to notice anything, appetite gone, countenance dark, often spotted or mottled, general dryness of the skin, mouth and tongue, tongue pointed, tremulous, red and fissured, and often considerable gastric irritation; incoherent talking, delirium, with constant picking at the bed-clothes, or feeling upwards with hands, as though desirous of grasping some object; sliding down to the foot of the bed or working across the bed upon the elbows, and often an effort to get out of bed; temperature from 103½° to 107°, and pulse often as high as 160 to the minute.

Sometimes these fevers will run unbroken for ninety days with but little change in temperature until large bed-sores are formed upon the hips and shoulder-blades, and with every effort to prevent I have seen these perfectly bare.

One of the characteristic symptoms of this is the slow and sneaking manner of its approach. The patient is not well, neither is he sick for many days before he takes his bed. The rose-colored eruption that is common to this disease is not well marked in every case, being very extensive in some, not only covering the umbilicus, but spreading over body and limbs, and in others, but few spots to be found anywhere. The odor peculiar to this disease is only characteristic of one other disease that I have treated, and that is small-pox. When the foot is pressed upon a wasp there is emitted a peculiar odor, and this is the odor of small-pox and typhoid fever, much stronger in small-pox, but never to be mistaken in typhoid fever. But constipated typhoid fever should not be confounded with typhus fever, and to this end I will give the distinction most marked:

| TYPHOID. | TYPHUS. |
|---|---|
| Typhoid fever is a disease of summer and autumn. | Typhus, a disease of winter and spring. |
| First week may be complicated with pneumonia. | Not until 2d or 3d week does pneumonia appear. |
| All climates subject. | Only temperate or cold climates. |
| Most liable between the ages of ten and thirty years. | All persons over 25 years. |

| TYPHOID. | TYPHUS. |
|---|---|
| Sudamina, 6-8-10 day. | No sudamina. |
| Rose-colored eruption that disappears on pressure and returns immediately. | Mulberry patches that do not disappear on pressure and often turn to dark, permanent spots or patechiæ. |
| Constipation with soreness of bowels and seldom ends under 21st day—is often protracted to the 44th day. | Often ends on the 14th day and seldom ever goes over 19 days. |

In constipated typhoid fever the tongue on the 5th or 6th day often cleans off very red, not always fiery red, as in perforation of the bowel. Then again, by placing the ear to the bowels, the gas can be heard passing down the bowel, and in the gravest form of the fever, it stops, collects and distends the bowel, and then with a gurgling sound, and often pain, it passes lower, either to escape, which is a favorable symptom, or, as is more often the case, turns back to renew its same course of collection, distension and pain. There are other prominent symptoms that belong to this disease, such as irregularity of the heart's action, distorted vision, subsultus tendinium, etc., but what I have enumerated is enough.

### PROGNOSIS.

In days gone by we were told that it was always unfavorable; but while it causes great anxiety to the patient and his friends, and oppressive watchfulness and care on the part of the physician, we can say prognosis is favorable in typhoid mitior and constipated typhoid fever, and unfavorable in typhoid gravior. It has been said that one in every three dies, but such is not my experience. It is but seldom one ever dies. Age has its influence, the old and very young requiring more sustaining treatment. The fat person, or very robust, fare much worse than others, and the danger to them is greater. Summer and fall less fatal than winter and spring; but when the fever season is winding up, we may well guard every point, for the danger is greater now than at any other time on account of some peculiar influence exerted upon the heart.

There are points just here to which I desire to direct your attention. When the tongue cleans off suddenly and assumes a fiery red, sleek and glazed appearance, perforation of the bowel is close at hand if not already present.

When the temperature goes down rapidly, and you see the face pale and anxious, cheeks flabby and drooping, respiration sighing, hemorrhage of the bowel is just back of this, and it is one of the most fatal complications of this fever.

When you see the thumb drawn down in the palm of the hand, the case will die.

Where many cases are sick in the same room, the next case occurring is apt to be of a violent type, more so at least than the cases that have preceded it.

When you see the corpus cavernosum penis hard and erect in any form of this disease, it is very favorable.

An ulcerated blister is said to be concomitant of this fever. Blistering is necessary in some cases, and when applied it seldom ever ulcerates, and when it does, even in extreme cases, recovery is almost certain.

I have never seen a case die in which there was strangury from any cause.

This disease is far more fatal among the poor than among the rich. When epidemic it takes up its line of march, and when the air is poisoned or polluted, when the streams reek with filth, it seems to be encouraged by winds and waters, and smites all alike.

CAUSE.

The medical profession for a long time has been greatly perplexed upon this point. Not satisfied with the cold assertion of malaria, for nearly two hundred years the germ theory has had its advocates. It is the predominating theory of the present day, and continued microscopic investigations are establishing it upon a better basis. This disease often is generated in situations where human beings are crowded together, and where ventilation is bad and food unwholesome Then we have seen these places free from the diseases during epidemics, as was the case this year at the different mining places in Jefferson county, where negroes are crowded, where there is a superabundance of filth, and the water is not good, any kind of food is eaten and not half prepared. Then we know it is also to be found on the tops of the mountains where the air is bracing, the water good as any that gushes from the earth, and the food wholesome, cleanly and properly prepared. Then we see it in the valleys and along the course of streams where malaria seems to be a privileged occupant. Many theories have existed in regard to the cause of this disease, but, like the kingdoms of the earth, have flourished, had their day, and passed away, and so it will be with the present one, like all the rest, leaving a light upon which the next generation may build a more acceptable and enduring one.

Malaria—what is it? Volumes have been written upon it, and yet it in material substance is unknown. It means "bad air," that is, air when cutaneously absorbed or inhaled by the lungs is capable of producing some material damage to a healthy subject, whether this result is accomplished by living beings capable of reproduction, or from injurious gases, the result of vegetable or animal decomposition or fermentation. But it has long been known that these same changes from health to disease are as much the effect of maludoria as malaria, and by that we mean water as well as bad air. But there is some consideration due to the manner of life, habits and constitution of the affected subject, as well as to extraneous influences. Can an atmosphere in close proximity with the surface of the earth be found free of wha may be termed impurities, or that will not leave its impress upon the brightest polished surface. Day has its sweetest breathing influence for man, or animals, and night develops the glories of the vegetable world. There is a power in this transition utilized for good or for evil. And where is the drop of water that is not filled with animal life? This imparts vitality to all that was pronounced good by an omnipotent Creator; and yet, where no power of assimilation exists in either animals or vegetables, they are overwhelmed by it, and diseases and death the result.

Susceptibility bears an important part in the transmission of disease, and the surroundings intensify the effect of slight differences in individual susceptibility. But we know that the persons most readily attacked are those most susceptible, and epidemics are often re-invigorated by such persons coming in contact with the generating influence.

Lancisi, in 1695, was the first to give life to the idea of materiality as the cause of fever, but not until within the last fifty years has the medical profession accepted the idea of a living germ being the cause of disease. 'Tis true that Linneas wrote of it in 1778, but little attention was given to the thoughts or ideas advanced by him. In 1848 my honored preceptor, Dr. John K. Mitchell, then the professor of practice of medicine in Jefferson Medical College, Philadelphia, gave us a lecture upon infusoria and animalculæ as the cause of fever, and an able paper afterwards published by him gave rise to farther investigation of the subject. In 1870 M. Bolestra, from his microscopical investigations and examinations of the water of the Pontine marshes in the vicinity of Rome, satisfied himself that he had found the malarial germ. A few years later (1875) Dr. Lanzi, of Rome, came to the same conclusion, the difference between them being in the pigment granules of the decaying Algæ, which he thought to be the same as the granules found in the spleen and liver of patients suffering from malarial poisoning. A little later, and Eklund, of Sweden, discovered a parasite in marsh mud, and seaweed from malarious localities, which, upon careful examination, he assumed to be the same as the parasite found in the blood of patients suffering from intermittent fever. In 1879, Klebs and Thommasi Crudeli made their experiments upon rabbits with the bacilli which they believed caused the malarial fever in man. This was injected subcutaneously, and they state as a result that the rabbits suffered with intermittent fever.

But this was not satisfactory, as by the experiments of Sternberg and others the same result was not obtained.

Following these are Marchiafava and Cuboni, in Italy, who thoroughly investigated the blood of persons who died with malarial fever, and in this they found spores and bacilli, which they were satisfied were the same as the bacilli of Klebs and Thommasi Crudeli. But this was done with post-mortem blood and was not satisfactory. Others have failed to find them in the blood of living patients. But in 1881 Laveran discovered a very different parasite, which he believed to be the true malarial germ. This was confirmed by Richard, who found the Laveran parasite in the blood of persons sick with malarial fever; yet, with this advancement in the way of the cause of disease, while there is no room for retreat, there has not been a satisfactory conclusion stamped upon the mind of the medical profession.

Murchison holds the position that typhoid fever is the result of the fermentation of fæces, healthy or unhealthy. I am satisfied that this was the cause of the epidemic of this fever this year in the southwest portion of Jefferson county. On both sides of Valley creek, from near its source to its mouth, the fever existed. This is a mountainous section, and this stream runs off rapidly, but in its course it is checked by mill-dams and natural dams. It has its origin at and near Birmingham, and into it all the garbage and sewer water and filth of every kind were emptied, and from it there arose an exceedingly disagreeable effluvia that were greatly increased during the summer, especially whenever there was a heavy rainfall. The sickness was confined almost entirely to this stream. The same cause does not seem to exist everywhere nor every year. In some portions of the State, when the fever season is unusually dry, the number of cases of typhoid fever is greatly increased. Such, at least, was the case in Marengo county, Alabama, from 1849 to 1857. The past season has been unusually wet with us, and we have had an epidemic of this fever, from which we naturally draw the inference that both wet and dry seasons are favorable to its development. Why may we not as reasonably say that cells under these extremes die and develop germs, the result of this fever, as to say that germs from extraneous sources develop or originate the fever? As far as the development has been made, it has been shown that there is a germ peculiar to each disease, whether the germ is the creature of the disease or the disease the work of the germ. Then why not each virulent form of disease produce its own germ? If we go to the vegetable kingdom, the tree that is bruised externally will always show it by its drooping leaves or dying branch, and if cut in deep its circulation is not only interrupted, but it soon withers and dies, and yet, under another kind of influence, its entire heart or inner body becomes diseased, rots out and it dies. All this the result of diseased cells. Why not so with man? Then again, if we take the known parts of earth upon which we dwell we find within itself waste, disease, decay, in the mineral and vegetable kingdom peculiar to each. Why not so in man and the entire animal kingdom? He is but a world of living animalcules only waiting development, and will manifest themselves after death, if not before. While I fully and firmly believe in the germ theory, I believe that there exist in every man cells that can be made to develop the same kind of germs that exist in the decay of the vegetable world.

May there not be such a thing as insolation or specific action, the result of solar heat, that would develop these same cells into a thing of life, that, like the vultures, would prey upon Prometheus' bowels. Our summer heat is very great and very great must be the influence exerted by it upon the nerve centres and the blood.

[To be continued.]

